# Computer tomographic assessment of gastric volume in major trauma patients: impact of pre-hospital airway management on gastric air

**DOI:** 10.1186/s13049-020-00769-y

**Published:** 2020-07-28

**Authors:** Thomas Mitteregger, Philipp Schwaiger, Janett Kreutziger, Herbert Schöchl, Daniel Oberladstätter, Helmut Trimmel, Wolfgang G. Voelckel

**Affiliations:** 1 Departement of Anaesthesiology and Intensive Care Medicine AUVA Trauma Centre Salzburg, Academic Teaching Hospital of the Paracelsus Medical University, Dr.-Franz-Rehrl-Platz 5, 5010 Salzburg, Austria; 2 Department of Anesthesiology and Critical Care Medicine, Medical University, Innsbruck, Austria; 3 Ludwig Boltzmann Institute for Experimental and Clinical Traumatology, AUVA Trauma Research Centre, Vienna, Austria; 4 Wiener Neustadt General Hospital, Department of Anaesthesiology, Emergency and Critical Care Medicine, and Karl Landsteiner Institute of Emergency Medicine, Wiener Neustadt, Austria; 5 University of Stavanger, Network for Medical Science, Stavanger, Norway

**Keywords:** Airway management, Pre-hospital intubation, Emergency room intubation, Gastric volume, Computer tomographic volume rendering, Major trauma

## Abstract

**Background:**

Gastric dilation is frequently observed in trauma patients. However, little is known about average gastric volumes comprising food, fluids and air. Although literature suggests a relevant risk of gastric insufflation when endotracheal intubation (ETI) is required in the pre-hospital setting, this assumption is still unproven.

**Methods:**

Primary whole body computed tomographic (CT) studies of 315 major trauma patients admitted to our Level 1 Trauma Centre Salzburg during a 7-year period were retrospectively assessed. Gastric volumes were calculated employing a CT volume rendering software. Patients intubated in the pre-hospital setting by emergency physicians (PHI, *N* = 245) were compared with spontaneously breathing patients requiring ETI immediately after arrival in the emergency room (ERI, *N* = 70).

**Results:**

The median (range) total gastric content and air volume was 402 (26–2401) and 94 (0–1902) mL in PHI vs. 466 (59–1915) and 120 (1–997) mL in ERI patients (*p = .*59 and *p = .*35). PHI patients were more severely injured when compared with the ERI group (injury severity score (ISS) 33 (9–75) vs. 25 (9–75); *p = .*004). Mortality was higher in the PHI vs. ERI group (26.8% vs. 8.6%, *p = .*001). When PHI and ERI patients were matched for sex, age, body mass index and ISS (*N* = 50 per group), total gastric content and air volume was 496 (59–1915) and 119 (0–997) mL in the PHI vs. 429 (36–1726) and 121 (4–1191) mL in the ERI group (*p = .*85 and *p = .*98). Radiologic findings indicative for aspiration were observed in 8.1% of PHI vs. 4.3% of ERI patients (*p = .*31). Gastric air volume in patients who showed signs of aspiration was 194 (0–1355) mL vs. 98 (1–1902) mL in those without pulmonary CT findings (*p = .*08).

**Conclusion:**

In major trauma patients, overall stomach volume deriving from food, fluids and air must be expected to be around 400–500 mL. Gastric dilation caused by air is common but not typically associated with pre-hospital airway management. The amount of air in the stomach seems to be associated with the risk of aspiration. Further studies, specifically addressing patients after difficult airway management situations are warranted.

## Background

A full or distended stomach is frequently observed in the primary radiologic assessment of trauma patients [1, 2]. Moreover, it is undisputed that trauma patients must always be considered to be at risk for aspiration. In a study measuring gastric volumes in injured patients by abdominal computed tomography (CT), volumes ≥700 cm^3^ were found to be associated with a 1.5 higher likelihood of pneumonia [3]. Unfortunately, the aforementioned study did not discriminate between food, fluids and air. The latter is of particular interest, because patients requiring pre-hospital airway management might be at risk of gastric ventilation and subsequently passive regurgitation. Among all major airway complications during intubation, aspiration is the most common cause of death [4]. Besides airway management complications, comprising inappropriate bag-mask-ventilation during induction of anaesthesia [5, 6], high flow oxygen mask breathing and distress might further contribute to aerophagia [7]. Presently, there is no conclusive evidence whether and to which extend pre-hospital airway management is associated with gastric dilation caused by air insufflation. Accordingly, we sought to retrospectively assess and quantify the gastric volume (comprising food, fluids and air) in severely injured trauma patients admitted to a Level 1 Trauma Centre by CT volume rendering. The primary goal of this study was to verify or reject the hypothesis that pre-hospital endotracheal intubation is associated with gastric insufflation and subsequently higher gastric air volumes assessed by the primary whole body CT-scan. As a secondary endpoint, we sought to evaluate if aspiration or death is associated with a higher amount of gastric content and / or air. Thus, we focused on severely injured patients intubated on scene or immediately after arrival in the emergency room.

## Methods

Retrospective analysis of trauma patients admitted to the Austrian Workers’ Compensation Board (AUVA) Level 1 Trauma Centre Salzburg emergency room between 2010 and 2017 with an Injury Severity Score (ISS) ≥9, and the need for ≥24 h intensive care treatment. Patients who have been intubated in the pre-hospital setting by emergency physicians (PHI), and patients intubated immediately after admission to the emergency room (ERI) were included. Of all patients matching the inclusion criteria, the primary whole-body CT scans, typically taken within 20 min after arrival, were further analysed. The gastric content was subsequently measured employing a new volume rendering software (syngo.via™, SIEMENS Healthineers, Erlangen, Germany), which has an accuracy of 85–100%.

### Setting

Austria in general, and Salzburg in particular relies on a professional physician-staffed ground and air rescue service. The Salzburg Trauma Network ensures that major patients are directly transported to one of the two Level 1 Trauma Centres by either ground ambulance (EMS) or helicopter emergency service (HEMS). The AUVA Trauma Centre Salzburg is a certified Level I trauma centre downtown Salzburg, Austria, participating in the German Trauma Registry Database. On average, 150 major trauma cases are admitted per year. Of all major trauma patients, comprehensive in-hospital data is available, thus allowing an insight in patient care and outcome assessment.

### Data collection

**Fig. 1:**
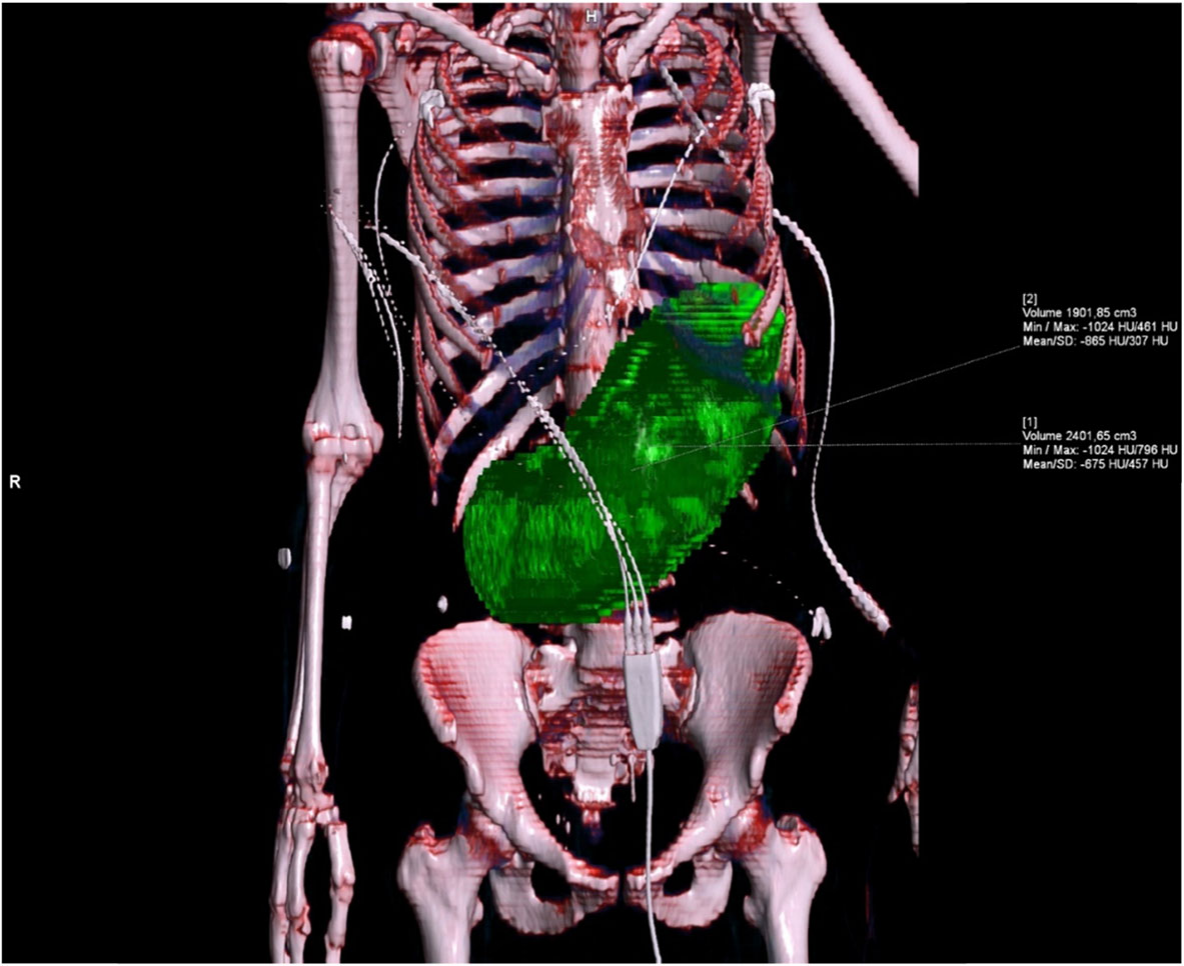
Example of gastric volume rendering in a patient with massive gastric dilation

Study patients were identified by extracting the Salzburg Trauma Centre cases from the German Trauma Registry Database. All patients matching the inclusion criteria were subsequently analysed employing the patient data management system (PDMS) COPRA 6™ (Berlin, Germany), and the AUVA electronic clinical information system. All trauma patients included underwent primary whole-body CT examination. CT scans are stored in an electronic radiography picture archiving and communication system (PACS) and were available for 3-dimensional assessment employing the aforementioned volume rendering software. Figure 1 shows an example of gastric volume rendering. Data were subsequently anonymized, entered in an MS Excel sheet (Microsoft, Redmond, WA, USA) and stored on data protected institutional hardware. All data obtained was handled according to current data protection guidelines as defined in the General Data Protection Regulation (EU-GDPR) allowing the processing of personal data necessary for the purposes of management of health care systems (Art. 9.2). The obligations for all persons involved in data processing are defined within our institution by the data protection officer and formally acknowledged. The ethical committee of AUVA declared the study unproblematic and granted permission (No. 21/2019).

### Matched pairs analysis

Since airway management problems comprising oxygenation, intermittent bag-valve-mask ventilation or tracheal intubation might be strongly influenced by the physiologic status and constitution of the patient, an additional matched pairs analysis was performed. Patients were matched according to sex (m/f), age (+/− 5 years), BMI (+/− 5%) and ISS (16–24 / > 24). Thus 50 patients in each group were identified and subsequently compared.

### Analysis of stomach volume and incidence of aspiration

In order to address a possible relationship between gastric air volume and aspiration, we identified all patients with radiographic signs of aspiration in the primary lung CT scans from both groups. Calculated gastric volumes were subsequently compared with the data from patients without aspiration.

### Statistical analysis

Normal distribution was tested using the Shapiro Wilks (SW) test. Continuous variables were expressed as median and range. Between-group differences for continuous variables were tested with Mann-Whitney U test. Categorical variables were analysed using the Chi-Square and Fisher exact test. In addition, we performed a simple logistic regression analysis in order to assess whether the dichotomous variable aspiration (yes or no) was predicted by the independent variable total gastric volume (food, fluids and air) or total gastric air alone. For the latter comparison an independent calculation was done, thus we sought to minimize the risk for collinearity. Statistical calculations were performed using GraphPad Prism 5.03 (GraphPad Software, La Jolla, CA, USA). The level of significance was set at *p* < .05.

## Results

368 of 946 patients identified in the Trauma Registry data base required immediate airway management: 258 (71%) were intubated during the pre-hospital phase and 110 (29%) within the first 60 min after admission to the emergency room (ER) because of the severity of their injuries or critical condition. When measurement of gastric content was inconclusive due to severe abdominal trauma or when death occurred before CT scanning, patients were excluded. Thus, 245 PHI and 70 ERI patients were included in the study. A typical CT volume rendering picture is depicted in Fig. 1.

### Demographics of PHI and ETI patients

There was no difference between groups concerning age and gender. Median injury severity score was significantly higher in PHI vs. ERI patients, which can be explained by the higher amount of brain trauma patients in the PHI group (*p* = .004; Table 1).

**Table 1:** Demographics of the study population

	*PHI* number§ or median (range)*	*ERI* number§ or median (range)*	*p value*
Number of patients§	245	70	–
Age*	46 (13–91)	50.5 (14–84)	NS
Gender			
Male§	191 (78%)	53 (76%)	NS
Female§	54 (22%)	17 (24%)	NS
Body mass index*	24.4 (16.4–39.2)	25.2 (20.5–42.4)	< 0.05
Injury Severity Score*	33 (9–75)	25 (9–75)	< 0.05
Abdominal trauma§	67 (27%)	31 (44%)	

### Total gastric content and air volume in PHI vs. ERI patients

Median (range) overall gastric content comprising fluids, food and air was comparable between groups with 402 (26–2401) mL in PHI vs. 466 (59–1915) mL in ERI patients (*p* = .59). Median (range) air volume was 94 (0–1902) in PHI vs. 120 (1–997) mL in ERI patients (*p* = .35; Table 2). Thus, pre-hospital airway management was not associated with higher gastric air volumes.

**Table 2:** Total gastric content, air volume and injury severity score in PHI vs. ERI patients

	PHI *median (range)*	ERI *median (range)*	*p-value*
Total gastric volume mL	402 (26–2401)	466 (59–1915)	0.59
Air volume mL	94 (0–1902)	120 (1–997)	0.35
ISS	33 (9–75)	25 (9–75)	0.004

### Total gastric content and air volume in a matched-pairs PHI vs. ERI patients

After matching for sex, age, body mass index and ISS, 50 patients in each group were identified. Median (range) overall gastric content in PHI vs. ERI 496 (59–1915) mL vs. 429 (36–1726) mL (*p* = 0.86), while median (range) air volume was 119 (0–997) mL vs. 121 (4–1191) mL (*p* = .98).

### Total gastric content and air volume in patients with aspiration

We observed more CT findings such as bronchial thickening, bronchiolectasis, centrilobular nodules, ground-glass opacities, atelectasis, consolidation or air trapping indicative for aspiration in PHI vs. ERI patients (8.1% vs. 4.3%; *p* = .32). Patients with vs. without signs of aspiration were more severely injured as indicated by the median (range) ISS (38 (7–75) vs. 29 (9–75); *p* = 0.03)).

Although overall gastric volume did not differ in patients with vs. without CT signs of aspiration (366 (26–2030) mL vs. 424 (56–2402); *p* = .71), we noted a tendency towards higher gastric air volumes in patients with signs of aspiration (194 (0–1355) mL vs. (98 (1–1902) mL; *p* = .08; Table 3).

**Table 3:** Total gastric content, air volume and injury severity score in patients with or without signs of aspiration

	Aspiration *median (range)*	No Aspiration *median (range)*	*p-value*
Total gastric volume mL	366 (26–2030)	424 (56–2402)	0.71
Air volume mL	194 (0–1355)	98 (1–1902)	0.08
ISS	38 (7–75)	29 (9–75)	0.03

In a simple logistic regression analysis, gastric air content but not total gastric volume was predictive for aspiration in PHI patients (*p* = .044), and in all intubated patients (PHI and ERI; *p* = .012; OR = 10,013).

### Impact of total gastric content and air volume on mortality

PHI patients had a higher mortality when compared with the ERI group (26.8% vs. 8.6%; *p* = 0.001). However, there was no difference in median (range) overall gastric content (492 (26–1402) vs. 408 (36–2030) mL; *p* = .17) and air volume (115 (0–1902) vs. 96 (1–1565) mL (*p* = .11) between patients who died vs. those who did not.

### Proximity to the hospital and decision making for PHI vs. ERI

To test the hypothesis that decision making for PHI might be influenced by the expected transport time, and thus eventually be postponed when the hospital is nearby, we compared transport times for PHI and ERI groups. No significant difference was observed between PHI vs. ERI patients (19 (13–26) vs. 18 (10–21) minutes (*p =* 0,1)).

## Discussion

In order to verify or reject the hypothesis that pre-hospital airway management is typically associated with gastric insufflation and subsequently gastric distension, we assessed gastric air volumes in 315 major trauma patients admitted to our Level 1 trauma centre. Interestingly, the amount of air in the stomach was comparable between patients undergoing tracheal intubation in the pre-hospital setting and patients transported spontaneously breathing to the emergency room. Thus, emergency airway management on-scene appears to be done proficient in our study population. Nonetheless, higher gastric air volumes were associated with more CT findings indicative for aspiration.

Our finding of a median total gastric volume of approximately 450 mL support the dogma that trauma patients must be considered as non-fasted [5, 8, 9], and therefore to be at risk for aspiration when airway management is required. In a study measuring gastric volumes in injured patients by abdominal computed tomography, volumes ≥700 cm^3^ were found to be associated with a 1.5 higher likelihood of pneumonia [3]. While, the aforementioned study did not discriminate between food, fluids and air, the CT volume rendering software employed in our study enabled us to calculate the total gastric content and air volume. The latter is of particular interest, because patients requiring pre-hospital airway management might be at risk of gastric ventilation and subsequently passive regurgitation. Although gastric distension in trauma patients has been observed in the primary radiologic assessment of trauma patients [1, 2], there is no literature addressing gastric air volume before and after emergency airway management. Interestingly, we did not observe a difference in total and gastric air volume when comparing PHI vs ERI patients. This can be explained by the fact that the EMS providers in our region are typically highly skilled anaesthesiologist being proficient in emergency anaesthesia and airway management. In other EMS settings results could be different, since literature suggests a higher rate of airway management problems in systems relying on paramedics or emergency medical technicians only [10]. Nonetheless, we noticed a few cases with excessive gastric distension up to 2.5 L and speculate that this might be caused by a difficult airway situation. Unfortunately, due to a lack of documentation provided by EMS, a detailed analysis of these cases was not possible. On the other side, we identified gastric air volumes in spontaneously breathing patients without pre-hospital intubation up to 1 L. This might be caused by a stress induced aerophagia or a positive airway pressure due to a high flow oxygen mask applied in major trauma patients with unprotected airways. Thus, the assumption that gastric air distension is more frequently observed in patients intubated on scene is not supported by our data.

It is undisputed that pre-hospital airway management is associated with a higher risk of complications, such as aspiration than intubation in a selective setting [11–16] [17, 18]. We found CT signs such as ground-glass opacities, atelectasis or consolidation indicative for aspiration in 8.1% of all patients in the PHI vs. 4.3% in the ERI group. Interestingly, the median gastric air volume in our study was approximately 100 mL higher in patients with CT signs of aspiration when compared with patients without aspiration, while total gastric volume did not differ. This is in accordance with the findings of Destrebecq et al. who showed that high volumes of air in the stomach significantly increased the risk of a ventilator associated pneumonia in intensive care patients, while gastric residual volumes were not associated with the incidence of pneumonia [19]. Another reason for aspiration might be the severity of trauma. Although all patients included in our study suffered a life-threatening injury, the median ISS value was significantly higher in patients with CT signs of aspiration. Interestingly, we did not find a difference in total gastric and air volume in patients who died during the course of treatment. Hence, there was no association between gastric dilation and death in our study.

Some limitations of the present study must be noted. First, due to the retrospective nature of our investigation, we are not able to report on pre-hospital airway management problems such as the need for intermitted mask ventilation or short episodes of oesophageal intubation. Unfortunately, information was found to be spare on the EMS reports, which must be considered as a major weakness of this study. Second, the difference in sample size between the PHI and ERI group is significant. Thus, we were not able to demonstrate a statistically significant difference in gastric air volume in patients with or without signs of aspiration, although our data suggests a certain correlation. Third, although the margin of error associated with the CT volume rendering software is expected to be less than 10%, this might also have an impact on our results. Finally, we believe that there is a major impact of quality of EMS service provided in our region. Thus, in other settings, results might differ.

## Conclusions

In major trauma patients, overall stomach volume deriving from food, fluids and air must be expected to be around 400–500 mL. Gastric dilation caused by air is common but not typically associated with pre-hospital airway management. The amount of air in the stomach seems to be associated with the risk of aspiration. Further studies, specifically addressing patients after difficult airway management situations are warranted.

## Data Availability

The datasets generated during and/or analysed during the current study are available from the corresponding author on reasonable request.

## Abbreviations

BMI: Body Mass Index
CT: Computer Tomography
EMS: Emergency Medical Service
ER: Emergency Room
ERI: Emergency Room Intubation
ETI: Endotracheal Intubation
HEMS: Helicopter Emergency Medical Service
ISS: Injury Severity Score
PACS: Picture archiving and communication system
PDMS: Patient Data Management System
PHI: Pre-Hospital Intubation
